# Early-life cortisol exposure modulates habituation to human disturbance in fallow deer fawns

**DOI:** 10.1098/rsbl.2026.0416

**Published:** 2026-10-14

**Authors:** George B. Brown, Dómhnall J. Jennings

**Affiliations:** School of Biological Sciences, Queen's University Belfast, Belfast, UK

**Keywords:** habituation, play, experience, learning, cortisol, fallow deer

## Abstract

The survival of vulnerable juveniles depends on how they respond to threat, yet the escape behaviour of individuals often differs despite similar exposure to stimuli. These differences may arise from interactions between experience, early-life physiology and developing behaviours such as play. Using flight initiation distance (FID) trials from fallow deer fawns (*Dama dama*) in an urban parkland, we investigated whether FID was associated with pedestrian exposure, locomotor play and cortisol extracted from neonatal hair. Fawns with lower cortisol showed shorter FIDs as prior pedestrian exposure increased, consistent with habituation to repeated low-risk human presence. By contrast, fawns with higher neonatal hair cortisol showed longer FIDs with increasing pedestrian exposure, suggesting maintained or increased sensitivity to disturbance. Accumulated locomotor play was not associated with FID, indicating that any benefits of play may be expressed in escape performance rather than the decision of when to flee. These results indicate that early-life physiological state influences how juveniles respond to repeated human exposure, with consequences for individual development of tolerance and escape behaviour. Understanding the physiological basis of habituation in young animals may help predict which individuals are most vulnerable to disturbance in human-dominated landscapes.

## 1. Introduction

Survival to reproductive maturity is a prerequisite for fitness [1], but small size and limited experience often make neonates vulnerable to predation [2]. Selection should therefore favour the early development of antipredator behaviours, including hiding, avoidance and escape [3,4]. Nevertheless, juveniles can differ markedly in how these responses develop, partly because individuals vary in early experience, physiological state and inherited or maternal effects [5–8]. For example, exposure to low-risk stimuli can vary markedly among juveniles and may generate individual variation in escape behaviour through divergent habituation or sensitization trajectories [9]. However, whether juvenile escape responses are shaped by exposure history alone, or also by physiological and behavioural differences, remains poorly understood.

Glucocorticoids are steroid hormones that help mediate vertebrate stress responses by mobilizing energy during challenges such as predator encounters [10]. As circulating glucocorticoids (cortisol in large mammals) accumulate in growing keratinized tissues, neonatal hair cortisol concentrations can provide an integrated measure of early-life glucocorticoid exposure [11,12]. This early exposure can in turn alter how individuals release glucocorticoids to later challenges, although the direction of this effect may depend on the timing and context of exposure [13,14]. Because glucocorticoids also influence learning, memory and attention to threat [15,16], neonatal cortisol exposure could affect how juveniles assess repeated disturbance. Such assessment is central to habituation, because repeated exposure to a stimulus does not necessarily reduce responses unless individuals learn that the stimulus poses little or no risk [17]. Exposure to glucocorticoids in early life may affect this process, influencing whether individuals attenuate, maintain or increase responses to repeated stimuli [17–19]. This could, therefore, partly explain why juveniles differ in their habituation to repeated low-risk disturbance.

Because escape decisions depend on the perceived costs and benefits of remaining versus fleeing [20], escape behaviour may also be influenced by juvenile play in addition to exposure history and endocrine state. Locomotor play comprises repeated, voluntary movements, such as running, leaping and bounding, that occur outside an immediate functional context [21]. This form of play has been proposed to support antipredator responses by developing the motor and behavioural skills needed during escape [22], through motor skill acquisition, refinement of neuromuscular coordination, preparation for unpredictable events or self-assessment of physical ability [23–27]. Juvenile survival has been positively associated with play in brown bears (*Ursus arctos*) [28,29], and mountain goats (*Oreamnos americanus*) [30], although whether this is a consequence of improved escape ability, altered escape decisions or alternative mechanisms remains unclear. Evidence from juvenile Belding's ground squirrels (*Urocitellus beldingi*) shows that social play, including locomotor manoeuvres, predicts motor development, boldness–exploration and faster escape from unfamiliar arenas, but also greater distances to detect and flee from approaching humans [25,31–33]. Together, these findings suggest that play may shape how juveniles assess approach risk, but its effects on escape distance may depend on whether play primarily increases tolerance of approach or promotes caution.

To estimate how disturbance exposure, early-life glucocorticoids and juvenile behaviour influence escape responses, we studied flight initiation distance (FID) in juvenile fallow deer fawns (*Dama dama*) in an urban parkland. Fallow deer are a popular park species where they can experience high levels of anthropogenic disturbance [34]. After birth, fawns remain concealed in vegetation before joining maternal groups at around three weeks of age, when they may experience sustained exposure to humans and increased opportunities to play with conspecifics [34,35]. Because fawns’ first sustained exposure to anthropogenic disturbance occurs when they enter maternal groups, this system allows individual responses to human presence to be tracked during development. In addition, fawns develop a full coat before birth, allowing cortisol extracted from neonatal hair to index early-life glucocorticoid exposure [12,36]. This is likelyto generate substantial individual variation in exposure to disturbance, habitat use, and opportunities for play. Together, these features make fallow deer well suited for testing how early-life experience, behaviour and developmental physiology relate to the emergence of flight responses [37,38].

We predicted that fawns with greater previous exposure to human presence would have shorter FIDs, consistent with habituation to repeated low-risk disturbance [17]. We also predicted that higher neonatal hair cortisol concentrations would be associated with longer FIDs reflecting increased sensitivity to perceived risk associated with early-life glucocorticoid exposure [39]. Because glucocorticoids can influence learning and threat assessment, we tested whether neonatal hair cortisol modified the relationship between previous human exposure and FID [15,16,40]. Specifically, this interaction tested whether behavioural responses to repeated disturbance differed according to neonatal glucocorticoid exposure. For play, we tested two alternative predictions. If locomotor play enhances motor competence, emotional regulation or self-assessed escape capacity, then fawns spending more time in play should tolerate closer approach and show shorter FIDs [23,27]. Conversely, if play promotes caution towards approaching stimuli, fawns that spend more time playing should initiate flight at greater distances and show longer FIDs [32,33].

## 2. Material and methods

### (a) Study site and population

The study was conducted in Phoenix Park, Dublin, a 707 ha public park of grassland and woodland, supporting approximately 600 free-roaming fallow deer. Two cohorts of fawns, in 2022 and 2023, respectively, were studied from July to late October. Annually, during the fawning months (late May–June), the park authorities permit researchers to locate and fit uniquely coloured numbered/lettered ear tags to fawns to facilitate individual identification [41].

### (b) Observation protocol

Groups containing fawns were monitored opportunistically throughout the day from a distance of 50 m to minimize observer-induced disturbance [34]. Focal groups were observed for 1 hour; sessions occasionally terminated early if the group dispersed before 1 hour had elapsed. We used all-event sampling to record occurrences of locomotor play and human disturbance [42]. Locomotor play was quantified at the individual level by recording the occurrence and duration of locomotor play, including running, bounding, jumping, bucking, chasing and coordinated movements with or without conspecifics (supplementary material, §S1.1). Human disturbance was recorded whenever pedestrians came within 50 m of the deer group [34]. Disturbance was measured at the group level; therefore, each disturbance event was assigned to all identified fawns present in the observed group. Groups were treated as distinct when separated by approximately 50 m [43]. Fawn sex was recorded after the study based on the development of antler pedicles and groin hair in males [34]. See tables S1.3.1–S1.3.3 in the supplementary material for summaries of observation effort by public-access category and habitat, and of locomotor play according to pedestrian presence.

### (c) Flight initiation distance

FID was measured as the distance at which a focal fawn moved away from an approaching researcher [44]. All trials were conducted by a single researcher wearing standardized clothing [45]. Focal fawns were selected opportunistically from peripheral resting individuals at the edge of groups before the approach began. Fawns facing into the group or resting with their head hidden were not approached. From a starting distance of approximately 100 m, the observer approached the focal fawn directly using regular strides at a constant walking pace. FID was recorded as the observer–fawn distance when the focal fawn first moved away from its resting position, measured using a rangefinder (Hawke Endurance LRF 700 OLED). For each trial, we recorded whether the group was in an area accessible to the public or restricted from public access, habitat type as open pasture or woodland, and group size as small (2–20 deer), medium (21–50 deer), large (51–100 deer) or very large (>100 deer). In total, 180 FID trials were recorded from 96 fawns. FID trials were conducted separately from group observation sessions. Some fawns were tested repeatedly in the analysed dataset, with repeat trials separated from 39 minutes to 77 days (median = 21.4 days); same-day repeat trials occurred in one fawn only in the final dataset.

### (d) Prior experience

To assess how prior experience influenced fawn FID, we calculated cumulative, observation-adjusted histories of locomotor play and pedestrian exposure for each fawn before each FID trial. Daily observation effort was calculated for each fawn from behavioural census records as the time the fawn was present and observable during monitored group sessions, excluding periods when it was not observed, had left the group, or the session was cut short. Locomotor play was summarized as the total daily duration of locomotor play behaviours. Pedestrian exposure was recorded at the group-observation level as the number of pedestrians involved in disturbance events and assigned to all identified fawns present in that group. Daily values were accumulated across the season and divided by cumulative observation time, producing cumulative rates of locomotor play duration and pedestrian exposure per observed hour. These variables were lagged by one day before being joined to the FID dataset, ensuring that each FID trial was associated only with experience accrued up to the end of the previous day.

### (e) Early-life cortisol

Hair was clipped from the ventral abdominal region of fawns during tagging in May–June 2022 and 2023, stored in paper envelopes at room temperature in the dark, and later analysed for cortisol. Cortisol extraction followed protocols previously developed and validated for fawn hair from this population [7,12]. Briefly, cleaned hair samples were washed, dried, extracted in methanol, evaporated under nitrogen, reconstituted and assayed using a commercial cortisol ELISA kit. Cortisol concentrations were corrected for starting hair mass and expressed as pg mg⁻¹ hair. Assay precision was acceptable, with intra- and inter-assay coefficients of variation of 9.54% and 12.17%, respectively [46]. Cortisol was quantified for 133 fawns. See the supplementary material, §S1.4, for the full protocol.

### (f) Statistical analysis

We analysed FID using Bayesian mixed-effects models in brms v 2.23.0 [47], with a lognormal likelihood to account for the positive, right-skewed distribution of flight distances. The model included neonatal hair cortisol, cumulative locomotor play experience rate (CLE; s h⁻¹ observed), cumulative pedestrian exposure rate (CPE; pedestrians h⁻¹ observed), day of study and group size as fixed effects. Group size was coded ordinally from small to very large (1–4). We included a neonatal hair cortisol × CPE interaction to test whether early-life glucocorticoid exposure modified the relationship between prior pedestrian exposure and FID. Fawn identity was included as a random intercept to account for repeated observations. Continuous predictors were standardized to mean zero and unit variance before analysis. To ensure cumulative experience predictors were based on sufficient prior observation time, we included only FID trials from fawns with at least 3 hours of focal observation accumulated before the day of the FID trial. After applying this prior-observation threshold and excluding observations with missing neonatal hair cortisol or covariate data, the primary dataset comprised 133 FID observations from 73 fawns.

Models were fitted using weakly informative regularizing priors (supplementary material, §S2.1), with convergence assessed using trace plots, effective sample sizes and rank-normalized $\hat{R}$ diagnostics [48]. Effects are reported as posterior medians with 95% credible intervals; effects were considered meaningful if at least 95% of the posterior distribution was on one side of zero. Sensitivity models tested whether the results were robust to stricter prior-observation thresholds, additional covariates omitted from the primary model to avoid over-parameterization (cohort, fawn sex, location, habitat and wind speed; see supplementary material, §S1.2), broader prior specification, and exclusion of the second FID trial from the single fawn tested twice on the same day.

To assess whether cortisol accounted for variation in fawn FID, we fitted an additional model which excluded both neonatal cortisol and its interaction with cumulative pedestrian exposure. We then descriptively compared the individual-level random-intercept variance between models with and without the cortisol terms (supplementary material, §S2.5). Full details of the statistical analysis are provided in the supplementary material, S2.

## 3. Results

In total, 180 FIDs were recorded from 96 fawns (mean = 31.4 m, s.d. = 11.3 m). After excluding observations with missing neonatal hair cortisol data, or less than 3 hours of prior focal observation, 133 FIDs from 73 fawns were retained for analysis (mean = 32.4 m, s.d. = 11.46 m). This meant 73.9% of the total FIDs were retained across 76% of individual fawns.

Fawns with higher neonatal hair cortisol had longer FIDs (*β* = 0.101; 95% CrI, 0.047–0.16; figure 1; supplementary material, figure S3.1). There was no clear independent effect of cumulative pedestrian exposure on FID when cortisol was held constant (*β* = −0.009; 95% CrI, −0.066 to 0.05; figure 1). However, the relationship between pedestrian exposure and FID interacted with neonatal cortisol, as indicated by a positive cortisol × pedestrian exposure interaction (*β* = 0.055; 95% CrI, 0.011–0.1; figures 1 and 2). Thus, cumulative pedestrian exposure was associated with longer FIDs among fawns with higher neonatal cortisol, whereas similar exposure was associated with shorter FIDS among fawns with lower neonatal cortisol.

**Figure 1.**
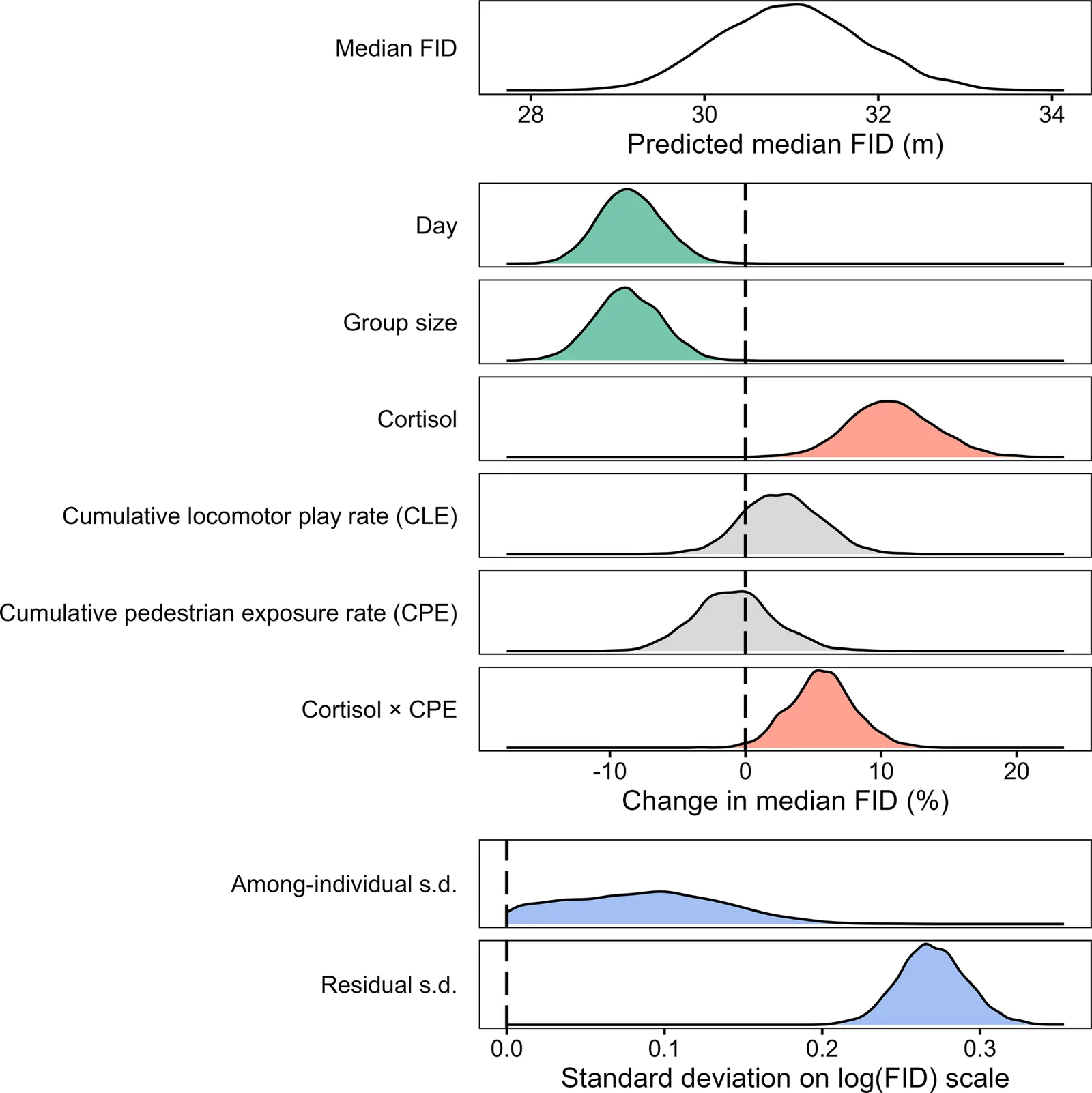
Posterior distributions from the FID model. The intercept is shown as predicted median FID (m). Fixed effects are shown as percentage change in median FID for a 1 s.d. increase in each predictor. Green indicates meaningful negative effects and red indicates meaningful positive effects, where 95% of the posterior lay on one side of zero. Grey indicates non-meaningful effects. Variance parameters are shown as standard deviations on the log-FID scale in blue.

**Figure 2.**
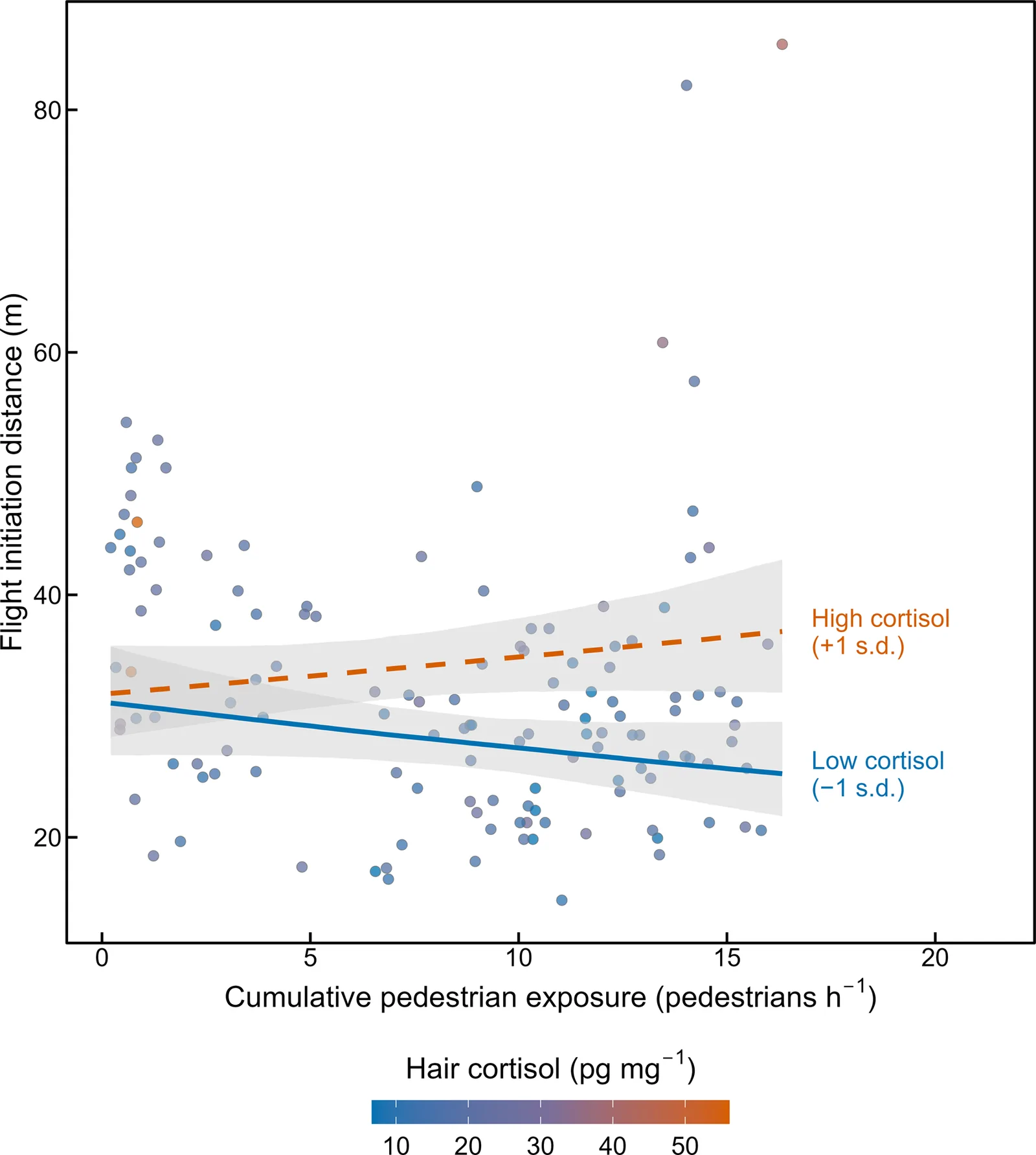
Relationship between cumulative pedestrian exposure (CPE) and FID as a function of neonatal hair cortisol. Points show observed FIDs. Lines show model-fitted FID for fawns with low (−1 s.d.) and high (+1 s.d.) neonatal hair cortisol, with shaded areas representing 95% credible intervals.

FID decreased with day of study (*β* = −0.089; 95% CrI, −0.138 to −0.037; figure 1) and fawns in larger groups had shorter FIDs (*β* = −0.091; 95% CrI, −0.144 to −0.039). There was no clear association between cumulative locomotor play experience and FID (*β* = 0.026; 95% CrI, −0.027 to 0.08; figure 1). The posterior median standard deviation of the individual random intercept was 0.086 (95% CrI, 0.005–0.18; variance = 0.0075), compared with a residual standard deviation of 0.27 (95% CrI, 0.23–0.31), indicating that substantial unexplained variation remained within individuals. However, when cortisol and its interactions were excluded, the individual random-intercept standard deviation increased to 0.106 (95% CrI, 0.008–0.208; variance = 0.011). Thus, including these terms was associated with a 31.8% reduction in unexplained among-individual variance, although uncertainty in the variance estimates was substantial (supplementary material, table S3.7).

The main effects identified in the primary model were retained across all sensitivity analyses, including models with stricter prior-observation thresholds, additional covariates broader prior specification, and exclusion of the single same-day repeat FID observation. Full sensitivity outputs are reported in the supplementary material, S3.

## 4. Discussion

Variation in escape behaviour is a critical determinant of survival [20] and is associated with a range of environmental, behavioural and physiological parameters [49]. However, the endocrine mechanisms underlying flight remain poorly understood, particularly outside adult life stages [37,40], and the role of locomotor play as training for predator escape has also received little attention [50]. Therefore, we tested whether prior pedestrian exposure, neonatal hair cortisol and accumulated locomotor play predicted FID in fallow deer fawns living in an urban park [34]. Our results suggest that early-life glucocorticoid levels modify how fawns respond to repeated human presence, whereas accumulated locomotor play was not associated with FID.

Human presence can elicit negative physiological and behavioural responses in free-ranging animals [51,52]. However, where anthropogenic contact is frequent and benign, such as in urban parkland, repeated exposure to humans should result in a decline in behavioural responding [9,53]. Our understanding of how habituation is affected by cortisol in free-ranging juveniles is sparse, although experimentally increased exposure to cortisol during the larval stage leads to attenuated habituation in zebrafish (*Danio rerio*) [54]. Here, we demonstrate that the relationship between pedestrian exposure and FID depended on neonatal hair cortisol. Specifically, fawns with lower neonatal hair cortisol tended to show shorter FIDs as pedestrian exposure increased, yet fawns with higher neonatal hair cortisol showed the opposite pattern, consistent with the idea that early-life glucocorticoid exposure can influence later learning, risk assessment or responsiveness to stress [18,54]. Consistent with this interpretation, the model without the cortisol terms had a greater posterior median remaining of the among-individual variance in FID, yet uncertainty around the variance estimates was large.

To our knowledge, this study is among the first to show that neonatal hair cortisol is associated with individual differences in habituation-like responses to human presence in free-ranging juveniles. However, the mechanisms underlying this association and the sources of variation in neonatal hair cortisol remain unclear. Experimental work indicates that acute glucocorticoid responses during encounters with novel threats can contribute to subsequent learning and memory [18]. Our results extend this work by suggesting that cortisol exposure integrated before the study period was associated with later differences in how fawns adjusted their FIDs as their experience with pedestrians accumulated. One possibility is that early-life glucocorticoid exposure shapes responses to later challenges, including acute glucocorticoid release [13,14], and may thereby influence subsequent learning. Variation in neonatal hair cortisol may itself reflect the fawn's exposure to stressors before capture, prenatal maternal effects or postnatal maternal influences mediated through maternal condition or behaviour. Our observational study cannot distinguish among these pathways. However, future studies combining repeated hormone measurements from mothers and offspring with experimental manipulation of disturbance could help resolve the underlying mechanisms and determine whether behavioural differences associated with neonatal cortisol persist over time. Nonetheless, our results help explain why repeated human exposure may produce habituation in some animals but sensitization in others [9]. Such state-dependent variation is relevant to urban wildlife management and release programmes, in which individuals may differ in how they adjust to novel environments and human disturbance [55,56].

We found no evidence that accumulated locomotor play was associated with escape decisions, indexed through FID. FID is thought to be an economic decision based on perceived risk and the costs of remaining versus fleeing [20], not a direct measure of acceleration, manoeuvrability or escape success. Therefore, if play does improve these abilities, but ability does not influence consequential decisions during escape [57], we are unlikely to detect an effect. Instead, future research should explore the possibility of measuring components of flight replicated during locomotor play behaviour, such as changes in direction and trajectory [58].

Our findings contrast with previous research in juvenile Belding's ground squirrels, in which play with conspecifics was associated with greater caution towards approaching humans, and experimental disruption of play reduced the developmental increase in distances to notice and flee from a human intruder [32,33]. One possibility is that the presence of conspecifics during play adds an external stimulus in which to monitor, engage and respond to. Such experiences may shape behavioural flexibility, coping style and threat assessment more directly than the performance of escape-like manoeuvres during solely locomotor play. Although our measure included locomotor play with and without conspecifics, future work separating different forms of play, including play-fighting or other reciprocal social play, may better reveal whether play influences escape decisions rather than escape performance.

In conclusion, experimental manipulation of cortisol has been linked with both habituation and sensitivity to risk-taking [36,59]. We extend these findings by showing that neonatal hair cortisol is associated with variation in subsequent habituation-like responses to human proximity in free-ranging juveniles. Thus, we show that risk-taking, habituation and escape decisions in a vulnerable prey species are shaped by interactions between environmental experience and developmental physiological state. Escape responses are economic decisions in which misjudged estimates of danger can incur costs through lost opportunities for maintenance activities [20]. However, whether divergent responses to human proximity ultimately will lead to differences in life-history outcomes remains to be determined. Ultimately, understanding wildlife responses to threats and human presence requires a broader focus than measuring environmental parameters alone. It requires recognition that animals may encounter the same world in different physiological states, which shape the developmental pathways of fear and tolerance.

## Supplementary Material



## Data Availability

Data are located in the supplementary material and are entitled ‘Data—Brown & Jennings 2026’. The associated code that uses these data is entitled ‘Code—Brown & Jennings 2026’. Information regarding the data can be found in the README file ‘README—Data—Brown & Jennings 2026.csv’. Supplementary material is available online [60].
