# Supplementary material for: Early-life cortisol exposure modulates habituation to human disturbance in fallow deer fawns
Source: Biol Lett. 2026 Oct 14;22(10):20260416. doi: 10.1098/rsbl.2026.0416 (PMC13682556; doi:10.1098/rsbl.2026.0416)
Supplement: Supplementary file 1 [file rsbl.2026.0416.zip › rsbl.2026.0416/rsbl20260416_si_001.docx]

**Early-life cortisol exposure modulates habituation to human disturbance in fallow deer fawns**

George B. Brown^1^ & Domhnall J. Jennings ^1^

^1^School of Biological Sciences

Queen’s University, Belfast,

19 Chlorine gardens,

BT9 5DL, Belfast

Ireland

**Supplementary material 1 – Further details: Materials and methodology**

1.1 Locomotor Ethogram

 Ethogram for recognising locomotor and social locomotor play behaviours in fallow deer fawns. These behaviours include vigorous movements such as running with stochastic and sudden changes in direction and gait either alone or with a conspecific

| **Behaviours** | |
| --- | --- |
| Gallop | A fast gait with independent cycling of limbs |
| Bound/jump | A jump in which front limbs are lifted first followed by the hindlimbs |
| Signal bound | A high jump with an exaggerated arch in back and forward limbs extended in front and hind limbs extend back |
| Stot | All limbs contact ground almost simultaneously following suspension |
| Stot-like jump | From standing, all four limbs depart the ground and land almost simultaneously |
| Turn | A directional change in locomotory play measured in increments of 90° |
| Zigzag | Quick movements from side to side during galloping or trotting |
| Buck | An individual raises its hindlimbs and kicks upwards with its forelimbs in contact with the ground |
| Chase | An individual follows another during a fast gait, potentially following changes in the leader's direction |
| Coordinated turning | Individuals change direction in a coordinated manner (simultaneously or immediately after) relative to each other |
| Coordinated bounds | Individuals bound in a coordinated manner (simultaneously or immediately after) relative to each other |

Informed by the ethogram of play in white-tailed deer, *Odocoileus virginianus*, and mule deer, *Odocoileus hemionus* [1]

1.2 Additional variables

Maximum daily wind gust speed (mph) was extracted from the weather data at Dublin Airport, located approximately 10km from the study site (<https://meteostat.net/en/>), and matched by the day the FID trial took place.

The habitat of the fawn during the FID was recorded as being either in open pasture or woodland. The location of the group was classified as either accessible to the public or closed to public access, for example within the campus of Táilte Éireann. Fawn cohort referred to whether fawns were studied in 2022 or 2023.

1.3 Observation summaries

Table S1.3.1. Number of group-observation sessions and total observation hours recorded in publicly accessible areas and areas without public access in 2022 and 2023. Observation duration was calculated as the total time observing the group.

| **Cohort** | **Location** | **N** | **Hours** |
| --- | --- | --- | --- |
| 2022 | Publicly accessible | 254 | 235 |
| 2022 | No public access | 40 | 38 |
| 2023 | Publicly accessible | 188 | 171 |
| 2023 | No public access | 90 | 80 |

Table S1.3.2. Number of group-observation sessions and total observation hours recorded in open pasture and woodland habitats in 2022 and 2023. Observation duration was calculated as the total time observing the group.

| **Cohort** | **Habitat** | **N** | **Hours** |
| --- | --- | --- | --- |
| 2022 | Open pasture | 140 | 128 |
| 2022 | Woodland | 154 | 144 |
| 2023 | Open pasture | 159 | 143 |
| 2023 | Woodland | 119 | 108 |

Table S1.3.3. Group observation-level locomotor play according to pedestrian presence for each study year. N is the number of group-observation sessions. “Play recorded” is the percentage of sessions in which locomotor play occurred by at least a single fawn. Play rate was calculated as the total duration of locomotor play (s) summed across fawns divided by the total usable fawn-observation time (h) within each session and is presented as the median and interquartile range (IQR).

| Cohort | Pedestrians recorded | N | Play recorded | Median Play Rate [IQR] |
| --- | --- | --- | --- | --- |
| 2022 | No | 90 | 52.22 | 0.33 [0 - 2.28] |
| 2022 | Yes | 204 | 62.25 | 0.35 [0-1.4] |
| 2023 | No | 100 | 59 | 0.28 [0-1.64] |
| 2023 | Yes | 178 | 53.37 | 0.14 [0-0.89] |

1.4 Hair collection and cortisol extraction

Hair was clipped from the ventral abdominal region of fawns during annual fawn tagging in May–June 2022 and 2023 and placed in paper envelopes where it was stored at room temperature in the dark until extraction. Cortisol extraction followed the protocol previously developed and validated for fawn hair from this population [2,3], based on established hair-hormone extraction methods [4,5].

Hair samples were manually cleaned of visible debris with tweezers, washed sequentially with deionised water and isopropanol on a tube rotator, and dried in a fume hood. 20 mg of hair, or the minimum available mass when less than 20 mg was available, was sonicated in 1 ml methanol and incubated in the dark for 18 h at 50°C and 100 rpm. Samples were then centrifuged briefly, and 600 µl of supernatant was transferred to clean microcentrifuge tubes and evaporated under nitrogen gas at 38°C. Dried extracts were stored at −20°C until assay and reconstituted in methanol and assay diluent at a 1:9 ratio. Cortisol concentrations were quantified according to the manufacturer’s instructions using a commercial ELISA kit (Salimetrics, item no. 1-3002). To account for variation in sample mass, cortisol values were corrected for hair weight and expressed relative to the starting mass of hair analysed (e.g. pg/mg hair).

This assay has previously been validated using two independent pooled samples from fawns of the same age range, population, and hair collection technique as those in the present study, including linearity, parallelism, and spike-recovery tests [2,3]. To confirm input-mass linearity in the present sample set, we created a pooled hair sample from 16 individuals balanced across sex, cohort, and hair colour, and used this pool to generate input hair masses ranging from 1 to 60 mg. Measured cortisol values showed a strong linear relationship with input hair mass across the 5–60 mg range (linear regression in R: R² = 0.9867, residual standard error = 0.0219, P < 0.001), supporting this interval as the working range for analysis.

Intra- and inter-assay precision for the main assay run were estimated using triplicate measurements of a pooled quality-control sample included on each of the four analytical plates. This yielded intra- and inter-assay coefficients of variation of 9.54% and 12.17%, respectively. Because this quality-control sample was not available on the validation plate, we performed an additional bridging check using a second pooled sample that had been assayed in quadruplicate on both the validation plate and one analytical plate from the main assay run. This comparison yielded a between-plate coefficient of variation of 6.97%. The manufacturer reports antibody cross-reactivity of 19.2% with dexamethasone and <0.6% with all other tested steroids.

Cortisol was extracted for 80 females (30 from 2022 and 50 from 2023) and 53 males (29 from 2022 and 24 from 2023), mean cortisol concentration extracted was 18.99 pg mg⁻¹ (sd = 8.044, range = 6.709-56.069) (Figure S1). There was no significant effect of sex on cortisol (pg/mg) (ANOVA: F_1,129_ = 0.24, *P* = 0.623), nor an effect of cohort (F1,129 = 2.52, *P* = 0.115), and no sex x cohort interaction (ANOVA: F_1, 129_ = 0.04, *P* = 0.842).

Figure S1. Neonatal hair cortisol concentration for each individual fawn, with bars coloured by sex. Horizontal lines indicate sex-specific mean cortisol concentrations

**References**

1.	Carter RN, Romanow CA, Pellis SM, Lingle S. 2019 Play for prey: do deer fawns play to develop species-typical antipredator tactics or to prepare for the unexpected? *Anim. Behav.* **156**, 31–40. (doi:10.1016/j.anbehav.2019.06.032)

2.	Amin B, Jennings DJ, Smith AF, Quinn M, Chari S, Haigh A, Matas D, Koren L, Ciuti S. 2021 In utero accumulated steroids predict neonate anti‐predator response in a wild mammal. *Funct. Ecol.* **35**, 1255–1267. (doi:10.1111/1365-2435.13790)

3.	Mortlock E, English H, Börger L, Matas D, Koren L, Capellini I, Jennings D. 2025 Drivers of individual differences in the sleep behaviour of fallow deer neonates. *J. Anim. Ecol.* **94**, 449–461. (doi:10.1111/1365-2656.14247)

4.	Koren L, Mokady O, Geffen E. 2008 Social status and cortisol levels in singing rock hyraxes. *Horm. Behav.* **54**, 212–216. (doi:10.1016/j.yhbeh.2008.02.020)

5.	Moya D, Schwartzkopf-Genswein KS, Veira DM. 2013 Standardization of a non-invasive methodology to measure cortisol in hair of beef cattle. *Livest. Sci.* **158**, 138–144. (doi:10.1016/j.livsci.2013.10.007)
