# Supplementary material for: Early-life cortisol exposure modulates habituation to human disturbance in fallow deer fawns
Source: Biol Lett. 2026 Oct 14;22(10):20260416. doi: 10.1098/rsbl.2026.0416 (PMC13682556; doi:10.1098/rsbl.2026.0416)
Supplement: Supplementary file 1 [file rsbl.2026.0416.zip › rsbl.2026.0416/rsbl20260416_si_002.docx]

**Early-life cortisol exposure modulates habituation to human disturbance in fallow deer fawns**

George B. Brown^1^ & Domhnall J. Jennings ^1^

^1^School of Biological Sciences

Queen’s University, Belfast,

19 Chlorine gardens,

BT9 5DL, Belfast

Ireland

**Supplementary material 2 – Further details: Statistical Analysis**

All the analysis was conducted in R 4.5.1 [1].

**2.1 FID model priors**

We used weakly informative regularising priors for the primary model. Fixed effects were assigned normal(0, 1) priors. This centred effects around zero while allowing substantial multiplicative changes in median FID for a one standard deviation change in a continuous predictor. This centred effects around zero while still allowing large changes in median FID for a one-standard-deviation change in a predictor. The model intercept was assigned a Student’s t prior with three degrees of freedom, centred at log(30 m), with a scale of one. This centred the prior for the population-level median FID at 30 m, while allowing substantial uncertainty on the response scale. The standard deviation of the individual-level random intercept and the residual standard deviation were each assigned exponential(1) priors. These priors were chosen to provide weak regularisation given the sample size, while allowing biologically plausible effect sizes.

**2.2 Bayesian parametrisation**

All models were run with four chains of 8000 iterations each, including 4000 warm-up iterations; every fifth post-warm-up draw was retained. Convergence was assessed by inspecting trace plots, effective sample sizes and rank-normalised R̂ diagnostics [2].Posterior distributions were considered to have converged adequately when R̂ values were < 1.01 and effective sample sizes were >1000. Results are reported as posterior medians with 95% credible intervals. We interpreted effects as having directional support when at least 95% of the posterior distribution was on one side of zero.

**2.3 Predictor correlations**
We assessed pairwise correlations among cumulative locomotor play experience, cumulative pedestrian exposure, and day of study in the primary 3 h dataset. Correlations were weak between day of study and cumulative locomotor play experience (CLE; r = 0.13) and between day of study and cumulative pedestrian exposure (CPE; r = 0.10). CLE and CPE were weakly negatively correlated (r = -0.22).

**2.4 Sensitivity models**

We ran additional sensitivity models to assess the robustness of our modelling approach. The model presented in the main text included FID trials from fawns with at least 3 h of prior focal observation. Because rate-based cumulative predictors may be sensitive to the amount of prior observation, we refitted the primary model using stricter inclusion thresholds of at least 5 h and 10 h of prior focal observation before each FID trial. The 5 h threshold model included 127 FID observations from 70 individuals, whereas the 10 h threshold model included 103 FID observations from 64 individuals. These models used the same likelihood, fixed-effect structure, random-effect structure and priors as the primary model but had reduced sample sizes. Results are reported in Tables S3.2 and S3.3.

To assess whether inferences from the primary model were sensitive to the choice of weakly informative priors, we refitted the model using broader priors while retaining the same likelihood, fixed-effect structure, random-effect structure and standardisation of continuous predictors. Fixed effects were assigned normal(0, 10) priors. The intercept was assigned a Student’s t prior with three degrees of freedom, centred at zero with a scale of 10, providing a broad prior on the log-FID scale. The standard deviation of the individual-level random intercept and the residual standard deviation were each assigned exponential(0.5) priors. Results were qualitatively unchanged under these broader priors, indicating that the main conclusions were not driven by prior specification (Table S3.4).

To assess whether estimates for the primary predictors were sensitive to additional covariates, we fitted an additional model that included location, habitat, fawn sex, study year and maximum wind gust speed. These covariates were not included in the primary model to avoid over-parameterisation given the available sample size. This model used the same prior specification as the primary model. Results are reported in Table S3.5.

To assess whether the single same-day repeat FID trial influenced inference, we refitted the primary model after excluding the second FID trial from the only fawn tested twice on the same day. This removed one observation, leaving 132 FID observations from 73 individuals. Results were qualitatively unchanged (Table S3.6).

**2.5 Cortisol and individual variance**

To assess the degree to which cortisol explained individual variance in FID, we removed cortisol and associated interactions from the main model. The model contained the same likelihood structure, priors and data as the main model (133 FID observations from 73 individuals). We then descriptively compared the individual-level random-intercept variance between models with and without the cortisol terms. The Results are in Table S3.7.

**References**

1.	R Core Team. 2025 R: A Language and Environment for Statistical Computing.

2.	Vehtari A, Gelman A, Simpson D, Carpenter B, Bürkner P-C. 2021 Rank-Normalization, Folding, and Localization: An Improved Rˆ for Assessing Convergence of MCMC (with Discussion). *Bayesian Anal.* **16**. (doi:10.1214/20-BA1221)
