# Supplementary material for: Early-life cortisol exposure modulates habituation to human disturbance in fallow deer fawns
Source: Biol Lett. 2026 Oct 14;22(10):20260416. doi: 10.1098/rsbl.2026.0416 (PMC13682556; doi:10.1098/rsbl.2026.0416)
Supplement: Supplementary file 1 [file rsbl.2026.0416.zip › rsbl.2026.0416/rsbl20260416_si_003.docx]

**Early-life cortisol exposure modulates habituation to human disturbance in fallow deer fawns**

George B. Brown^1^ & Domhnall J. Jennings ^1^

^1^School of Biological Sciences

Queen’s University, Belfast,

19 Chlorine gardens,

BT9 5DL, Belfast

Ireland

**Supplementary material 3 – Further details: Results**

**Table S3.1. Results from the primary Bayesian mixed-effects model of juvenile flight initiation distance (FID).** FID was modelled using a lognormal error distribution. Continuous fixed effects were standardised to a mean of 0 and a standard deviation of 1 before analysis. The model included 133 FID observations from 73 individuals and included an individual-level random intercept to account for repeated observations. Only FID trials for which the focal fawn had accumulated at least 3 h of prior observation were included. Estimates are posterior medians with 95% credible intervals.

| Parameter | Median | 95% CI | Px | Rhat | ESS |
| --- | --- | --- | --- | --- | --- |
| **Intercept** | **3.434** | **3.382 , 3.487** | **0** | **1** | **3181** |
| **Cortisol (pg/mg)** | **0.101** | **0.047 , 0.159** | **0** | **1** | **3265** |
| Cumulative pedestrian exposure rate (CPE) | -0.009 | -0.066 , 0.05 | 38.3 | 1 | 3077 |
| Cumulative locomotor play experience rate (CLE) | 0.026 | -0.027 , 0.08 | 17.1 | 0.999 | 3141 |
| **Day of the study** | **-0.089** | **-0.138 , -0.037** | **0.1** | **0.999** | **3170** |
| **Group size** | **-0.091** | **-0.144 , -0.039** | **0.1** | **1** | **3372** |
| **Cortisol x CPE** | **0.055** | **0.011 , 0.1** | **0.9** | **0.999** | **3205** |
| ID | 0.086 | 0.005 , 0.182 | 0 | 0.999 | 1784 |

Figure S3.1. Posterior fitted flight initiation distance (FID) in relation to hair cortisol concentration, with cumulative pedestrian exposure rate, cumulative locomotor play experience rate, study day and group size held at their mean values. Points represent the 133 flight distances included in the main model. The solid line shows the population-level fitted effect, and the shaded ribbon represents the 95% credible interval.

**Table S3.2. Results from the 5 h observation-threshold sensitivity model of juvenile flight initiation distance (FID).** FID was modelled using a Bayesian mixed-effects model with a lognormal error distribution. Continuous fixed effects were standardised to a mean of 0 and a standard deviation of 1 before analysis. The model included 127 FID observations from 70 individuals and included an individual-level random intercept to account for repeated observations. Only FID trials for which the focal fawn had accumulated at least 5 h of prior observation were included. Estimates are posterior medians with 95% credible intervals.

| Parameter | Median | 95% CI | Px | Rhat | ESS |
| --- | --- | --- | --- | --- | --- |
| **Intercept** | 3.442 | 3.389 , 3.497 | 0 | 1 | 3050 |
| **Cortisol (pg/mg)** | **0.096** | **0.039 , 0.154** | **0.1** | **1** | **3106** |
| Cumulative pedestrian exposure rate (CPE) | -0.002 | -0.062 , 0.056 | 46.8 | 1.002 | 3113 |
| Cumulative locomotor play experience rate (CLE) | 0.028 | -0.026 , 0.082 | 14.4 | 0.999 | 3209 |
| **Day of the study** | **-0.104** | **-0.156 , -0.054** | **0** | **1.001** | **3038** |
| **Group size** | **-0.088** | **-0.142 , -0.034** | **0.1** | **1** | **3227** |
| **Cortisol x CPE** | **0.051** | **0.006 , 0.098** | **1.4** | **1** | **3161** |
| ID | 0.084 | 0.005 , 0.185 | 0 | 0.999 | 1965 |

**Table S3.3. Results from the 10 h observation-threshold sensitivity model of juvenile flight initiation distance (FID).** FID was modelled using a Bayesian mixed-effects model with a lognormal error distribution. Continuous fixed effects were standardised to a mean of 0 and a standard deviation of 1 before analysis. The model included 103 FID observations from 64 individuals and included an individual-level random intercept to account for repeated observations. Only FID trials for which the focal fawn had accumulated at least 10 h of prior observation were included. Estimates are posterior medians with 95% credible intervals.

| Parameter | Median | 95% CI | Px | Rhat | ESS |
| --- | --- | --- | --- | --- | --- |
| **Intercept** | **3.406** | **3.345 , 3.465** | **0** | **1** | **3060** |
| **Cortisol (pg/mg)** | **0.106** | **0.042 , 0.173** | **0.1** | **1** | **2705** |
| Cumulative pedestrian exposure rate (CPE) | -0.011 | -0.075 , 0.054 | 36 | 1 | 3180 |
| Cumulative locomotor play experience rate (CLE) | 0.017 | -0.045 , 0.08 | 29.6 | 1 | 2636 |
| **Day of the study** | **-0.07** | **-0.128 , -0.01** | **1** | **0.999** | **3166** |
| **Group size** | **-0.076** | **-0.135 , -0.018** | **0.7** | **0.999** | **3134** |
| **Cortisol x CPE** | **0.052** | **0.003 , 0.1** | **1.8** | **0.999** | **3124** |
| ID | 0.076 | 0.004 , 0.191 | 0 | 0.999 | 2064 |

**Table S3.4. Results from the broad-prior sensitivity model of juvenile flight initiation distance (FID).** FID was modelled using a Bayesian mixed-effects model with a lognormal error distribution. This model used the same structure and dataset as the primary model, including 133 FID observations from 73 individuals, with an individual-level random intercept to account for repeated observations. Only FID trials for which the focal fawn had accumulated at least 3 h of prior observation were included. Continuous fixed effects were standardised to a mean of 0 and a standard deviation of 1 before analysis. To assess prior sensitivity, this model was fitted with weaker priors than the primary model: fixed effects were assigned normal priors with a mean of 0 and standard deviation of 10, the intercept was assigned a Student’s *t* prior with 3 degrees of freedom, location 0, and scale 10, and both the individual-level random-intercept standard deviation and residual standard deviation were assigned exponential priors with a rate of 0.5. Estimates are posterior medians with 95% credible intervals.

| Parameter | Median | 95% CI | Px | Rhat | ESS |
| --- | --- | --- | --- | --- | --- |
| **Intercept** | **3.434** | **3.379 , 3.488** | **0** | **1.001** | **3193** |
| **Cortisol (pg/mg)** | **0.101** | **0.045 , 0.161** | **0.1** | **1** | **2646** |
| Cumulative pedestrian exposure rate (CPE) | -0.007 | -0.066 , 0.052 | 40.3 | 1.001 | 3133 |
| Cumulative locomotor play experience rate (CLE) | 0.026 | -0.027 , 0.082 | 16.7 | 1 | 3406 |
| **Day of the study** | **-0.089** | **-0.139 , -0.039** | **0.1** | **1.001** | **3232** |
| **Group size** | **-0.09** | **-0.144 , -0.038** | **0.1** | **1** | **3258** |
| **Cortisol x CPE** | **0.055** | **0.008 , 0.1** | **0.8** | **1** | **3132** |
| ID | 0.086 | 0.005 , 0.184 | 0 | 0.999 | 2201 |

**Table S3.5. Results from the fuller covariate sensitivity model of juvenile flight initiation distance (FID).** FID was modelled using a Bayesian mixed-effects model with a lognormal error distribution. This model used the same observation threshold as the primary model and included 133 FID observations from 73 individuals, with an individual-level random intercept to account for repeated observations. Only FID trials for which the focal fawn had accumulated at least 3 h of prior observation were included. Continuous fixed effects were standardised to a mean of 0 and a standard deviation of 1 before analysis. In addition to the predictors included in the primary model, this fuller model included maximum wind gust, pedestrian location, habitat, sex, and year. This model was used as a sensitivity analysis to assess whether focal estimates were robust to additional contextual covariates. Estimates are posterior medians with 95% credible intervals.

| Parameter | Median | 95% CI | Px | Rhat | ESS |
| --- | --- | --- | --- | --- | --- |
| **Intercept** | **3.416** | **3.284 , 3.557** | **0** | **1.001** | **3098** |
| **Cortisol (pg/mg)** | **0.095** | **0.036 , 0.152** | **0.2** | **1.001** | **2719** |
| Cumulative pedestrian exposure rate (CPE) | 0.015 | -0.054 , 0.082 | 32.7 | 1 | 2974 |
| Cumulative locomotor play experience rate (CLE) | 0.032 | -0.022 , 0.086 | 12 | 1.001 | 2956 |
| **Day of the study** | **-0.064** | **-0.121 , -0.008** | **1.6** | **1.001** | **3250** |
| **Group size** | **-0.062** | **-0.132 , 0.005** | **3.8** | **1** | **3132** |
| Maximum wind gust speed (m/s) | -0.036 | -0.087 , 0.017 | 9.1 | 0.999 | 3273 |
| Location (restricted) | 0.114 | -0.06 , 0.287 | 9.9 | 1.001 | 3122 |
| Habitat (woodland) | -0.038 | -0.15 , 0.074 | 24.5 | 1 | 3180 |
| Sex (male) | -0.044 | -0.158 , 0.063 | 21.8 | 0.999 | 3034 |
| Study year (2) | 0.03 | -0.083 , 0.14 | 30.5 | 1.001 | 3079 |
| **Cortisol x CPE** | **0.058** | **0.012 , 0.104** | **0.5** | **1** | **2985** |
| ID | 0.081 | 0.005 , 0.186 | 0 | 1 | 1796 |

**Table S3.6. Results from the same-day sensitivity Bayesian mixed-effects model of juvenile flight initiation distance (FID).** FID was modelled using a lognormal error distribution. Continuous fixed effects were standardised to a mean of 0 and a standard deviation of 1 before analysis. The model included 132 FID observations from 73 individuals, after excluding one FID observation recorded 39 minutes after a previous FID trial from the same fawn on the same day. An individual-level random intercept was included to account for repeated observations. Only FID trials in which the focal fawn had accumulated at least 3 h of prior observation were included. Estimates are posterior medians with 95% credible intervals.

| Parameter | Median | 95% CI | Px | Rhat | ESS |
| --- | --- | --- | --- | --- | --- |
| **Intercept** | **3.435** | **3.386 , 3.489** | **0** | **1** | **3110** |
| **Cortisol (pg/mg)** | **0.102** | **0.044 , 0.159** | **0.1** | **1** | **3293** |
| Cumulative pedestrian exposure rate (CPE) | -0.009 | -0.064 , 0.05 | 37.5 | 1 | 2861 |
| Cumulative locomotor play experience rate (CLE) | 0.026 | -0.029 , 0.081 | 17.1 | 1 | 3199 |
| **Day of the study** | **-0.089** | **-0.137 , -0.038** | **0.1** | **1** | **3157** |
| **Group size** | **-0.092** | **-0.145 , -0.038** | **0** | **0.999** | **2985** |
| **Cortisol x CPE** | **0.055** | **0.009 , 0.099** | **0.8** | **0.999** | **2873** |
| ID | 0.084 | 0.004 , 0.18 | 0 | 1 | 2017 |

**Table S3.7. Results from the Bayesian mixed-effects model of juvenile flight initiation distance (FID) with cortisol removed.** FID was modelled using a lognormal error distribution. Continuous fixed effects were standardised to a mean of 0 and a standard deviation of 1 before analysis. The model included 133 FID observations from 73 individuals. An individual-level random intercept was included to account for repeated observations, and cortisol related predictors were removed from the model. Only FID trials in which the focal fawn had accumulated at least 3 h of prior observation were included. Estimates are posterior medians with 95% credible intervals.

| Parameter | Median | 95% CI | Px | Rhat | ESS |
| --- | --- | --- | --- | --- | --- |
| **Intercept** | **3.428** | **3.372 , 3.488** | **0** | **1** | **3151** |
| Cumulative pedestrian exposure rate (CPE) | 0 | -0.061 , 0.062 | 49.8 | 1 | 2950 |
| Cumulative locomotor play experience rate (CLE) | 0.043 | -0.013 , 0.099 | 7.1 | 1.001 | 3282 |
| **Day of the study** | **-0.106** | **-0.162 , -0.054** | **0** | **0.999** | **3387** |
| **Group size** | **-0.098** | **-0.153 , -0.042** | **0.1** | **1** | **3317** |
| ID | 0.106 | 0.008 , 0.208 | 0 | 1.002 | 1702 |
